# An approach to identify the novel miRNA encoded from *H. Annuus* EST sequences

**DOI:** 10.1016/j.gdata.2015.09.005

**Published:** 2015-09-09

**Authors:** Hemant Gupta, Tanushree Tiwari, Maulik Patel, Aditya Mehta, Arpita Ghosh

**Affiliations:** Xcelris Labs Ltd., Ahmedabad, Gujarat, India

**Keywords:** *Helianthus annuus*, MicroRNA, Expressed sequence tags, Circos plot, Target prediction, Phylogenetic analysis, Gene ontology, Novel miRNAs, miRBase, Viridiplantae

## Abstract

MicroRNAs are a newly discovered class of non-protein small RNAs with 22–24 nucleotides. They play multiple roles in biological processes including development, cell proliferation, apoptosis, stress responses and many other cell functions. In this research, several approaches were combined to make a computational prediction of potential miRNAs and their targets in *Helianthus annuus* (*H. annuus*). The already available information of the plant miRNAs present in miRBase v21 was used against expressed sequence tags (ESTs). A total of three miRNAs were detected from which one potential novel miRNA was identified following a range of strict filtering criteria. The target prediction was carried out for these three miRNAs having various targets. These targets were functionally annotated and GO terms were assigned. To study the conserved nature of the miRNAs, predicted phylogenetic analysis was carried out. These findings will significantly provide the broader picture for understanding the functions in *H. annuus*.

## Highlights

• MicroRNAs are new class of small RNAs with 22-24 nucleotide. They play significant roles in regulating the cell functions
• Here Insilco approach has been used for computational prediction of potential miRNAs and their targets from H. annuus ESTs
• This analysis will add a resource to small RNA repository and decipher the undiscovered information from ESTs
• 3 miRNAs were identified from which one is potential miRNA for H. Annus species,not yet submitted in miRBase version 21
• GO annotation of the targets is helping in getting the better insights of the targets predicted
• Circos plot representation of the targets and their respective miRNAs gives pictorial representation of the targets

## 1 Introduction

In plants, miRNAs play important roles in plant post-transcriptional gene regulation by targeting mRNAs for cleavage or repressing translation. miRNAs are the class of endogenous, small, non-coding, single stranded RNAs located mostly within non-coding regions of the genomes and usually transcribed from RNA polymerase II promoters [1], [2]. The generation of mature miRNA is a complicated enzyme catalyzed process, from the initial transcript pri-miRNA to the precursor (pre-miRNA) with a characteristic hairpin structure, then a miRNA duplex (miRNA:miRNA*). The last step is that it gets assembled to the RNA-induced silencing complex (RISC) to direct its activity on a target mRNA [3]. The action of this complex depends on the degree of base paired between miRNA and responsive elements, resulting in the translational repression of the target mRNA. The perfect pairing results in cleavage whereas imperfect ones lead to translational repression in plants [4]. miRNA genes represent about 1–2% of the known eukaryotic genomes and constitute an important class, who has an important role in regulating the several physiological processes at different levels [5].

The first plant miRNAs were identified in early 2002 from model plant *Arabidopsis thaliana*. Since then a large number of plant miRNAs have been submitted in miRBase Sequence database (http://www.mirbase.org). In case of the availability of whole genome sequence of an organism, a large number of computational approaches has been designed to search miRNA. The unavailability of whole genome sequences played a great limitation in this strategy of the miRNA identification [6], [7]. EST analysis is a powerful tool to identify miRNAs conserved among various plant species whose whole genome sequences are not available, and to study the conservation and evolution of miRNAs among different species. EST analysis strategies have been proven to be successful for the discovery of new miRNAs from various plant species. Thus, the Insilco based approaches are very useful for predicting novel miRNAs, which cannot be detected by the wet lab methods [8].

*Helianthus annuus* is recognized worldwide for its beauty; it is also an important source of food. *H. annuus* oil is a valued and healthy vegetable oil and *H. annuus* seeds are enjoyed as a healthy, tasty snack and nutritious ingredient to many foods. Thousands of microRNAs have been discovered in recent years but there have been only 6 novel miRNAs that have been reported for the *H. annuus* in the repository. Therefore, in the present study, we have used all known plant miRNAs from Viridiplantae to search the conserved *H. annuus* miRNA homologs in a publicly available EST database [9], [10], [11].

## 2 Results

**Fig. 1:**
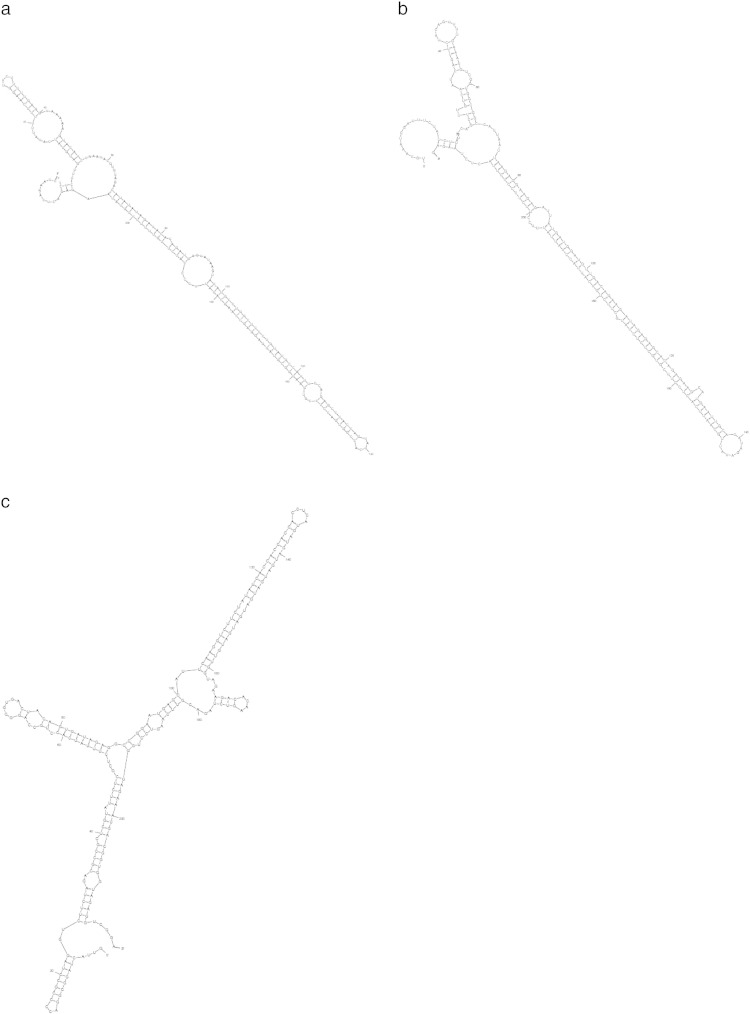
(A) Stem loop structure of predicted han-miR160a. (B) Stem loop structure of predicted han-miR156c. (C) Stem loop structure of predicted han-miR396.

A total of three potential miRNAs were identified with the predicted stem loop precursor structure from the publicly available EST database. There are large numbers of evidences stating the conserved nature of plant miRNAs [12]. This feature of theirs provides the powerful methods to identity the new miRNAs in plant species. After the removal of the repetitive miRNA sequences from the Viridiplantae group the remaining 4617 miRNAs were locally aligned against 134,475 EST sequences of *H. annuus* by using BLAST program with e-value 1000, percentage identity greater than 85, word size 7 and mismatch less than 4. The BLAST alignment detected the homology of Viridiplantae mature miRNA with the 20 sequences of *H. annuus* ESTs. The validation process for these miRNAs was initiated by secondary structure prediction using mfold web server. This is a very critical step in deciding the fate of the miRNAs, so the following important parameters were studied: 1) Minimum free energy (negative MFE), 2) adjusted minimal fold energy (AMFE) and 3) the minimal fold energy index (MFEI) were used. The secondary structure prediction filtered out 17 potential miRNA candidates and only three of them passed the criteria which are listed in Table 1 and their predicted secondary structures are shown in Fig. 1A–C. These potential candidates encode as han-miR160a, han-miR156c and han-miR396 families. The potential miRNAs had higher minimal folding free energy (MFE) and minimum free energy index (MFEI); the MEFI ranging between 0.65 to 0.85 and AU content of pre-miRNA within 30% to 70%.

**Fig. 2:**
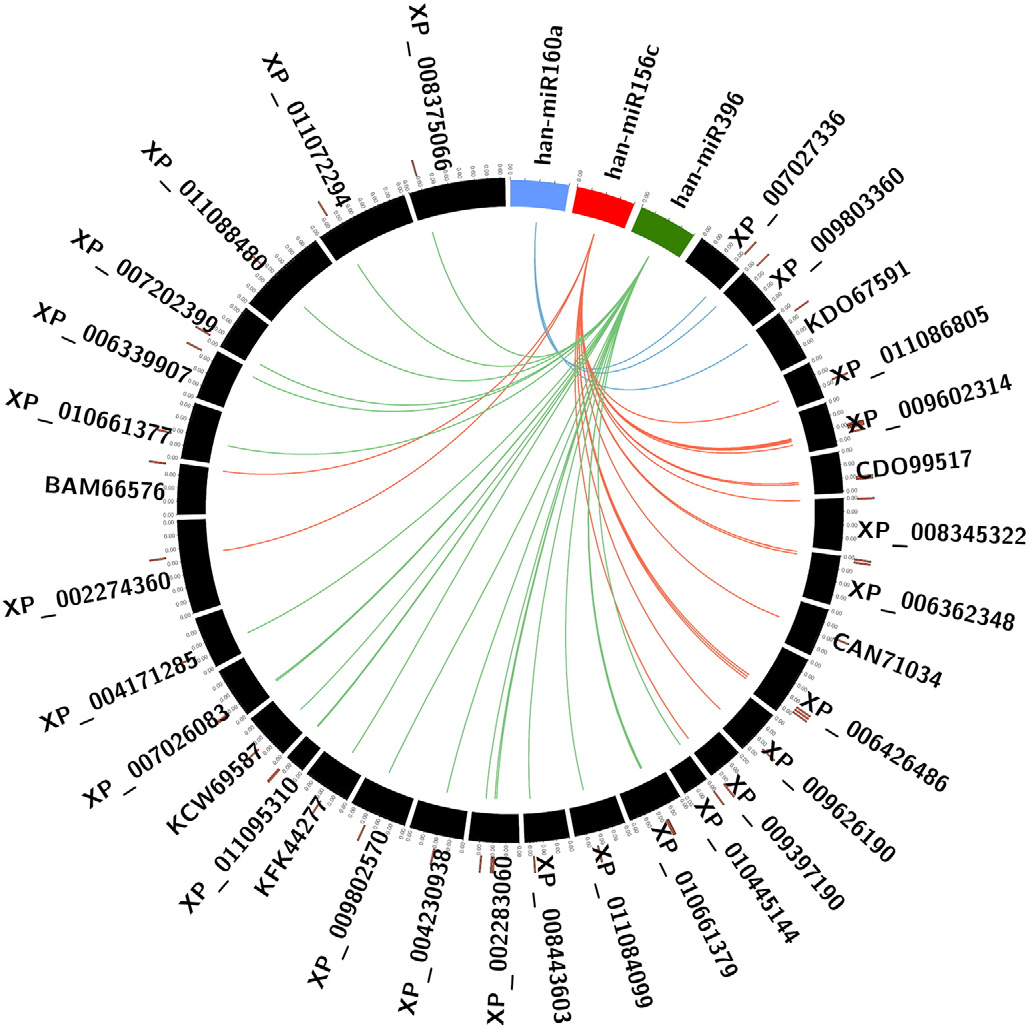
Circos plot between the three predicted miRNAs and their targets.

The expressions of miRNAs are regulated by a specific gene via hybridization on mRNA transcripts to promote RNA degradation, inhibit translation or both. To search the putative target genes of three putative miRNAs, a psRNATarget program with default parameters was used to predict the targets against the *H. annuus* ESTs and Unigenes [13], [14]. A total of 59 and 29 targets were predicted from ESTs and Unigenes respectively. The two miRNA families han-miR160a and han-miR156c show the complementarity with *H. annuus* miRNAs present in miRBase v21 but, han-miR396 is novel to its species and shows significant similarity with *Cynara cardunculus* species of the database. The targets of the three miRNAs were plotted against its targets using the circos plot as represented in Fig. 2.

**Fig. 3:**
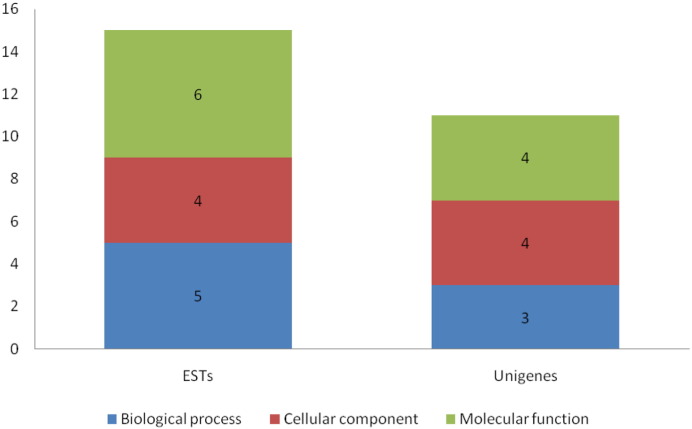
GO distributions of miRNA targets among the EST and Unigene data sets.

The targets were functionally annotated, followed by GO annotation. These miRNA targets belonged to a number of gene families that are involved in different biological functions. The miRNA family miR396 showed the highest 32 numbers of independent targets followed by other two families; simultaneously 8 targets were annotated for biological process and cellular component and 10 for molecular function from three miRNA families as concluded in Fig. 3.

**Fig. 4:**
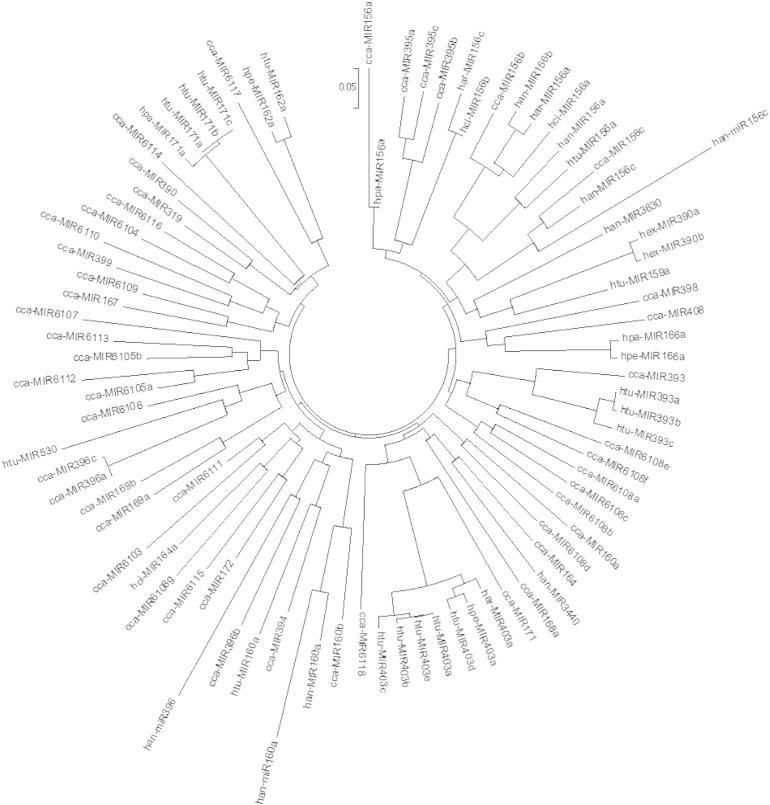
Phylogenetic analysis of pre-miRNAs sequences in different families.

The conserved nature of the plant miRNAs, at precursor levels among the distantly related plants was studied using MEGA 6 and a phylogenetic tree was generated as represented in Fig. 4. These results suggested that different miRNAs might evolve at different rates not only within the same plant species, but also in different ones. han-miR396 showed an unrelated evolutionary relationship with other miRNAs.

## 3 Discussion

Many recent studies have demonstrated that plant miRNAs are involved in many crucial metabolic activities [15]. The second important feature of the plant miRNAs is their conserved nature. Therefore, we have used all the previously known plant mature miRNAs from miRBase repository to search for homologs of miRNAs of *H. annuus* in the publicly available EST database [16]. By computational predictions, we found 3 miRNAs belonging to different miRNA families. The formation of the stem loop hairpin secondary structure is the critical step in miRNA maturation and is also the important characteristic of pre-miRNAs. To differentiate the miRNAs from other non-coding RNAs, minimum free energy is an important criterion that was considered. MFE determines the secondary structure of the nucleic acids [17], [18]. The lower the value of MFE, the higher the stability of secondary structure of corresponding sequence. The structures of the hairpin are shown in Fig. 1A–C.

To get insights of the key role played by the miRNAs in plant development and other activities, the targets were predicted. The targets were predicted using the computational approach [19]. It was seen that most of the targets were the genes coding for transcription factors and regulatory proteins involved in the transcription and folding of protein. In some cases it was seen that the miRNAs were having complementary relation with more than one regulatory target. These targets can be grouped based on their functions. The present research has been successful in finding one new miRNA family for *H. annuus* which is not deposited in miRBase repository followed by its target prediction, function and GO annotation.

## 4 Materials and methods

### 4.1 Reference data set of miRNA

To identify potential miRNAs, a total of 8496 miRNAs from group Viridiplantae were considered from publicly available database miRBase version 21. To remove the redundancy, the repeated miRNAs were clustered into single using cd-hit-v4.5.4 [20] with identity value 100. The data size almost got reduced to half and subsequently 4617 non-redundant unique miRNAs were obtained. These miRNAs were taken as a reference set of miRNA sequences.

### 4.2 *H. annuus* ESTs

A total 134,475 ESTs of *H. annuus* were extracted from the EST of Genbank nucleotide database and all of these sequences were screened against the known plant miRNAs for the homology.

## 5 Bioinformatics tools

The similarity search was carried out using BLAST-2.2.30 + program downloaded from the NCBI ftp site (ftp://ftp.ncbi.nih.gov/). miRNA precursor folding was performed by the mfold web server [21]. Those miRNAs which folded well were taken for target prediction using psRNATarget: a plant small RNA target analysis server. Simultaneously the conserved nature of miRNAs among the homologs was studied using MEGA 6. Circos plot was drawn to visualize the novel miRNAs and their corresponding targets [22].

### 5.1 Prediction of potential miRNAs

The sequences of the known plant miRNAs were used as query sequences for BLAST against the EST databases; with the parameter e-value being 10 and word match size between the query and database sequences is 7. Mature miRNA sequences should not be less than 18 nt and should be a maximum of 24 nt. The precursor sequences of 200 nt were extracted (100 bp upstream and 100 downstream to the BLAST hits) and used for hairpin structure prediction. If the length of EST sequence was less than 200 nt, the entire available EST sequence was used as a miRNA precursor sequence. These precursor sequences were aligned against nr database using BLASTX to remove the protein coding sequences. The non-protein coding sequences were used for hairpin structure prediction using mfold web server. Those meeting the following criteria were designated as miRNA homologs: (1) The RNA sequence folding into an appropriate stem-loop hairpin secondary structure, (2) a mature miRNA sequence located in one arm of the hairpin structure, (3) miRNAs having less than 6 mismatches with the opposite miRNA ∗ sequence in the other arm, (4) no loop or break in miRNA ∗ sequences, (5) predicted secondary structures with higher minimal folding free energy (MFE) and minimal folding free energy index (MFEI); the MEFI ranging between 0.65 and 0.85, and (6) the AU content of pre-miRNA within 30% to 70% [23].

### 5.2 Computational prediction of miRNA targets

MiRNA target prediction was performed by psRNATarget [11]. In this predicted miRNA sequences were analyzed against the unigenes and ESTs of *H. annuus* with default parameters including, maximum expectation threshold: 3, length for complementarity scoring (hsp size): 20, target accessibility — allowed maximum energy to unpair the target site (UPE): 25, flanking length around target site for target accessibility analysis 17 bp in upstream/13 bp in downstream and range of central mismatch leading to translational inhibition was set between 9 and 11 nt. After the removal of the repeated sequences, the potential target sequences were BLAST against protein databases to predict their function (identity > 25%). The functional annotation was followed by GO analysis, carried out using Blast2GO pro server [24].

### 5.3 Phylogenetic analysis of the new miRNAs

Considering that the sequences of miRNAs have been conserved in nature, the mature sequences of the novel and the known miRNAs in the same family were aligned and phylogenetically analyzed by MEGA 6 [25] to investigate their evolutionary relationships.

## Conflict of interest

The authors have declared that no competing interests exist.

## Figures and Tables

**Table 1:** Details of the predicted miRNAs from EST.

miRNAs	Mature sequence	Star sequence	LM	LS	LP	G + C	MFE	MFEI
han-miR160a	GCCUGGCUCCCUGUAUGCCA	UGGCGUAUGAGGAGCCAAGC	20	20	220	38.99	− 68.5	0.79857306
han-miR156c	UGACAGAAGAUAGAGAGCAC	GUGCUCUCUAUGCUUCUGUCA	20	21	220	38.18	− 71.7	0.85361208
han-miR396	AUUCAAGGUCUUCUAUAUCA	UGAUGAUGAUGAUGUUGG	20	18	220	50.45	− 72.6	0.65411298
